# Human DRG Glucocorticoid Receptor Profiling Reveals Targets for Regionally Delivered Steroid Analgesia

**DOI:** 10.3390/cells15030223

**Published:** 2026-01-24

**Authors:** Shaaban A. Mousa, Elsayed Y. Metwally, Xiongjuan Li, Sascha Tafelski, Oscar Andrés Retana Romero, Jörg Piontek, Sascha Treskatsch, Michael Schäfer, Mohammed Shaqura

**Affiliations:** 1 Department of Anaesthesiology and Intensive Care Medicine, Charité—Universitätsmedizin Berlin, Corporate Member of Freie Universität Berlin, Humboldt-Universität zu Berlin and Berlin Institute of Health, Charité Campus Benjamin Franklin, Hindenburgdamm 30, 12203 Berlin, Germany; elsayed.metwally@charite.de (E.Y.M.); sascha.treskatsch@charite.de (S.T.);; 2 Department of Anesthesiology, Shenzhen Second People’s Hospital, The First Affiliated Hospital of Shenzhen University, Health Science Center, Shenzhen 518035, China; 3 Department of Anaesthesiology and Intensive Care Medicine, Charité—Universitätsmedizin Berlin, Corporate Member of Freie Universität Berlin, Humboldt-Universität zu Berlin and Berlin Institute of Health, Charité Campus Mitte and Campus Virchow Clinic, Charitéplatz 1, 10117 Berlin, Germany; 4 Clinical Physiology/Nutritional Medicine, Department of Gastroenterology, Rheumatology and Infectious Diseases, Charité—Universitätsmedizin Berlin, 12203 Berlin, Germany

**Keywords:** glucocorticoids, mineralocorticoids, receptors, peripheral sensory neurons, pain, human, rats

## Abstract

**Highlights:**

**What are the main findings?**

- Glucocorticoid receptors (GRs) are the predominant corticosteroid receptors expressed in human dorsal root ganglia (DRG).
- GRs are preferentially localized to nociceptive DRG neurons, colocalizing with CGRP and only occasionally with GFAP of satellite glial cells.

**What are the implications of the main findings?**

- Preclinical and clinical data support a functional role for neuronal GR signaling in attenuating inflammatory and radicular pain, respectively.
- Mineralocorticoid receptor (MR) signaling emerges as a pronociceptive counterpart, highlighting receptor-selective modulation as a therapeutic strategy.

**Abstract:**

Corticosteroid receptor signaling in primary afferent neurons of the dorsal root ganglion (DRG) has emerged as a potential target to modulate nociception via genomic and nongenomic mechanisms shown in animal pain models. However, the expression landscape of glucocorticoid receptors (GRs) relative to mineralocorticoid receptors (MRs) in human DRG, their association with pain-related markers, and their functional relevance remain incompletely defined. We analyzed human and rat DRG by mRNA profiling and immunofluorescence confocal microscopy to assess GR/MR expression and complemented these studies with a clinical evaluation of neuraxial corticosteroid delivery. Here, GR transcripts in human DRG were the most abundant among corticosteroid receptor-related genes examined (including MR) and were observed alongside transcripts of pain-signaling molecules. Human DRG immunofluorescence analysis revealed substantial colocalization of GR with calcitonin gene-related peptide (CGRP), a marker of nociceptive unmyelinated C-fibers and thinly myelinated Aδ-fibers, as well as with gial fibrillary acidic protein (GFAP), a marker of satellite glial cells (SGCs), but minimal expression in myelinated neurofilament 200 (RT-200) immunoreactive (IR) human DRG neurons. In addition, GR immunoreactivity was primarily distributed to medium-diameter neurons (40–65 µm). Functionally, preclinical experiments showed that GR activation and MR blockade attenuate inflammatory pain via rapid, nongenomic neuronal mechanisms that counter an intrinsic mineralocorticoid receptor-mediated pronociceptive drive. Consistently, clinical analgesia over at least 3 months after transforaminal plus caudal epidural delivery of GR agonists in chronic radicular pain supports a functional role for neuronal GR signaling within spinal cord and DRG circuits. Together, these molecular, functional, and clinical findings identify GR as a key modulator of sensory neuron excitability and pain, highlight MR as a pronociceptive counterpart, and suggest that selectively enhancing GR signaling or inhibiting MR signaling may offer a potential strategy for improving corticosteroid-based analgesic therapies.

## 1. Introduction

Mineralocorticoids and glucocorticoids are key regulators of multiple mammalian systems including metabolism, immunity, cardiovascular function, reproduction, behavior, and cognition, through activation of their respective receptors, the MR and the GR [1]. For decades, systemic or local activation of the GR has been known to suppress inflammation [2], whereas MR activation has been implicated in pro-inflammatory and fibrotic processes, e.g., within the cardiovascular system [3,4,5]. Traditionally, these effects were attributed to intracellular receptor signaling processes that modulate gene transcription. However, accumulating evidence demonstrates rapid, nongenomic actions of mineralocorticoids with much faster onset [6,7,8,9]. Such nongenomic effects can trigger cellular inflammatory responses within 10–15 min [10] and, in peripheral sensory nerves, may contribute to the development of persistent pain [11].

Recent experimental work has identified both MR and GR predominantly on peripheral CGRP–IR nociceptors, with only sparse colocalization in spinal glia or astrocytes [9,12]. This distribution aligns with a functional role in modulating nociceptive behavior [9,12]. More recently, we showed that aldosterone is produced locally within peripheral sensory neurons and that intrinsic activation of neuronal MR sustains mechanical hypersensitivity during inflammatory pain [13]. In contrast, GR agonists engage a rapid, nongenomic, membrane-associated, G-protein-coupled mechanism that acutely inhibits pain [9].

Clinically, chronic back pain remains difficult to treat in patients who do not respond to medication or physiotherapy. Epidural steroid injection has emerged as a promising intervention for managing chronic back pain [14]. In animal pain models, steroids may act by interrupting nociceptive C-fiber signaling and by reducing perineural edema [15,16]. Although the precise mechanisms of epidurally administered steroids are not fully resolved, their effects likely involve activation of corticoid receptors within the spinal cord, nerve roots, and DRG.

A growing body of experimental evidence highlights the DRG as a central hub for pain transduction and relay to the central nervous system. Notably, one recent study has examined MR and GR in human DRG using in situ hybridization, reporting GR and MR expression over a broad range of nociceptive neurons [17]. To address remaining gaps in the anatomical, molecular, and functional characterization of glucocorticoid signaling in DRG, we extend the work of Qualls et al. (2025) [17] by systematically comparing GR expression patterns and localization in human and rat DRGs, as well as assessing their functional relevance across species. First, we quantified mRNA expression levels of GR and MR in human and rat DRGs and related these levels to the expression of well-established pain-associated ion channels and neuropeptides. These molecular findings were complemented by immunohistochemical analyses examining GR localization in relation to nociceptive markers (including CGRP and TrkA) and MR in human DRGs, with direct comparison to rat tissue. To explore functional implications, we evaluated the effects of exogenous activation and inhibition of GR and MR using selective agonists and antagonists in experimental models of inflammatory pain. Finally, to provide a translational perspective, we performed a secondary analysis of clinical data from patients receiving epidural steroid treatment for lumbar radicular pain, thereby linking experimental observations to human pain conditions.

## 2. Materials and Methods

### 2.1. Collection of Human and Rat DRG Tissue Samples

Frozen and paraformaldehyde-fixed human lumbar (L4) dorsal root ganglia (DRGs) were obtained from two young adult donors (female, 20 years; male, 22 years) who died as a result of traumatic accidents. Tissue was provided by AnaBios Corporation and Donor Network West (San Ramon, CA, USA). Lumbar DRGs were selected to facilitate direct comparison with rat experiments, and tissue from young adult donors was used to minimize the potential influence of age-related comorbidities on gene expression and histological outcomes. Rat DRG were collected from naïve, healthy Wistar rats (*n* = 6; 250–300 g). Immediately after procurement, DRG tissues were either snap-frozen in liquid nitrogen or fixed in 4% paraformaldehyde prior to shipment. Frozen DRG were bisected to generate two technical replicates for quantitative real-time PCR analyses (details provided below). The suppliers confirmed that informed consent and all required authorizations for the transfer and research use of donor tissues had been obtained. The study was conducted in accordance with the Declaration of Helsinki and was approved by the Charité Institutional Review Board (EA1/228/14). All animal procedures were approved by the competent authority in Berlin (LaGeSo; approval number G0024/14).

### 2.2. Experimental Animal Protocols

Adult male Wistar rats weighing 250–300 g (Janvier, Berlin, Germany; 6–8 animals per experimental group) were used for all experiments. Animals were briefly rendered unconscious using inhaled isoflurane anesthesia before induction of inflammation. Local inflammation was initiated by administering a single injection of 0.15 mL Freund’s complete adjuvant (FCA; Calbiochem, Darmstadt, Germany) into the plantar surface of the right hind paw (approved animal protocol G0024/14). This intervention consistently produces a unilateral inflammatory response, manifested by paw swelling and local hyperthermia, recruitment of multiple immune cell types, and an increase in mechanical pain sensitivity. The characteristics and reproducibility of this model have been well documented previously [18]. In this model of FCA-induced hind paw inflammation, the effects of subcutaneous administration of increasing doses of either the GR agonist dexamethasone (0.1–2 mg/kg) (Sigma-Aldrich, St. Louis, MO, USA), the GR antagonist mifepristone (1.0–4.0 mg/kg) (Sigma-Aldrich, St. Louis, MO, USA), the MR agonist aldosterone (0.5–2 mg/kg) (Sigma-Aldrich, St. Louis, MO, USA), the MR antagonist canrenoate K (10–40 mg/kg) (Sigma-Aldrich, St. Louis, MO, USA), or vehicle were evaluated. Dose ranges and the experimental time course were based on prior pilot studies. Mechanical paw pressure thresholds (PPTs) were assessed using a Randall-Selitto pressure apparatus (Ugo Basile SRL, Monvalle, Italy) at baseline and 15 min after drug administration. The force required to elicit paw withdrawal, i.e., paw pressure threshold (PPT) was determined as the average of three consecutive measurements separated by 10 s. To minimize order effects, the sequence of testing left and right paws was alternated across animals. Behavioral results are presented as mean ± SD of the percentage of maximum possible effect (%MPE), and are calculated as follows: %MPE = (PPT_post-injection_ − PPT_baseline_)/(70_control_ − PPT_baseline_) × 100, measuring increasing mechanical sensitivity relative to PPT values of control animals, i.e., 70 g. In all behavioral experiments, drugs were prepared by an independent experimenter (M.Sh.), and the examiner (X.Li) was unaware of the treatment each animal received; however, no formal randomization procedures were used to assign animals to experimental groups.

### 2.3. Quantitative TaqMan^®^ qPCR in Human and Rat DRG

Lumbar dorsal root ganglia (DRGs; segment L4) were rapidly dissected from human and rat tissue, snap-frozen in liquid nitrogen, and stored at −80 °C until processing. Total RNA was isolated from each DRG sample using a RNeasy kit (Qiagen, Hilden, Germany). For complementary DNA (cDNA) synthesis, equal amounts of total RNA from each sample were reverse-transcribed using commercially available reverse transcription kits (Takara, Göteborg, Sweden) optimized for human or rat RNA, respectively. The reverse transcription reaction was performed in a thermocycler under conditions recommended by the manufacturer, including an initial priming step, a reverse transcription incubation at elevated temperature to improve transcript coverage, and a final enzyme inactivation step. Resulting cDNA was diluted to a uniform working concentration prior to quantitative PCR analysis. 

Gene-specific primer pairs targeting glucocorticoid receptor (GR), mineralocorticoid receptor (MR), Nav1.8, Nav1.9, TRPV1, CGRP, Tac1, and the reference gene 18S rRNA were used (primer sequences are provided in Table S1). Primer specificity and amplification efficiency were validated prior to experimental use. TaqMan^®^ quantitative polymerase chain reaction (TaqMan^®^ qPCR) was performed using a real-time PCR detection system and an SYBR^®^ Green-based master mix, prepared according to the supplier’s instructions. Each reaction mixture contained diluted cDNA template, forward and reverse primers at optimized concentrations, SYBR^®^ Green master mix, and nuclease-free water in a final reaction volume suitable for the instrument used. 

Thermal cycling was conducted for a total of 40 amplification cycles. Each cycle consisted of an initial denaturation step at 95 °C for 15 s. Primer annealing and extension were carried out at 60 °C for 30 s for all target genes, with the exception of 18S rRNA, for which optimized conditions were applied. Fluorescence acquisition was performed at gene-specific temperatures slightly below the experimentally determined melting temperature (Tm) to enhance detection of specific amplicons (GR: 83 °C; MR: 84 °C; 18S: 88 °C). To confirm amplification specificity, melting curve analyses were performed at the end of each run, ensuring the presence of a single, gene-specific peak. All reactions were performed in technical triplicates for each human or rat tissue sample, and mean cycle threshold (Ct) values were calculated for subsequent analysis.

### 2.4. Immunohistochemistry in Human and Rat DRG

Human L4 DRG were obtained already fixed in 4% paraformaldehyde and washed in distilled water for 48 h. Rat DRG (*n* = 4–6) were collected from naïve Wistar rats (Janvier Labs) following deep isoflurane anesthesia and transcardial perfusion with 100 mL warm saline, followed by 300 mL 4% paraformaldehyde in 0.16 M phosphate buffer (pH 7.4). DRGs were dissected and post-fixed for 90 min. Human and rat samples were cryoprotected overnight at 4 °C in PBS containing 10% sucrose, rinsed in PBS, and stored at −80 °C. Tissue was sectioned at 10 µm and mounted on gelatin-coated slides. Every fourth section from each rat or human donor, with ≥10 sections per antibody, was processed for immunofluorescence. Slides were washed in PBS and incubated in freshly prepared sodium borohydride (1 mg/mL in PBS) for three 10 min cycles, followed by 60 min in blocking solution (PBS with 0.3% Triton X-100, 1% BSA, 10% goat serum, 10% donkey serum). Sections were incubated overnight with primary antibodies against GR [19] together with CGRP [20], TrkA [21], RT200 [22], MR [23] or GFAP [24] (Table S2). After PBS washes, secondary antibodies were applied (Alexa Fluor 594 donkey anti-rabbit or anti-mouse, Vector Laboratories; Alexa Fluor 488 goat anti-guinea pig or anti-mouse, Invitrogen). Nuclei were counterstained with DAPI (0.1 μg/mL, Sigma). Sections were mounted in Vectashield and imaged using a Zeiss LSM 510 confocal microscope equipped with argon (458/488/514 nm), HeNe 543 nm, and HeNe 633 nm lasers [20]. Imaging parameters (laser power, detector gain, brightness, contrast, scanning time, pinhole) were kept constant for each experimental set. Fluorescence was detected using a Plan-Neofluar 40×/1.3 NA oil objective. Quantification of immunoreactive profiles was conducted by blinded evaluator and followed established procedures [11,20]. In brief, cell soma diameter of immunostained DRG neurons was measured using Zeiss ZEN 3.8. High-resolution immunofluorescence images were acquired under identical microscope settings and analyzed in 2D view. Only neurons with clearly defined cell bodies and intact morphology were included. The maximal soma diameter (Feret diameter) was measured by drawing a straight line across the longest axis of each cell body; for irregularly shaped cells, two perpendicular diameters were measured and averaged. Pixel-to-micrometer calibration was verified using objective metadata. Measurements were automatically recorded in micrometers and exported for statistical analysis. Values are reported as frequency or ratio (in percent) and presented as means ± SD. Specificity controls included omission of primary and/or secondary antibodies to assess tissue autofluorescence and nonspecific binding (Supplementary Figure S1). Validation of antibody specificity has been reported previously. The polyclonal rabbit anti-GR antibody (gift from M. Kawata, Kyoto) has been validated in COS-1 cells with and without GR expression [12]. The monoclonal mouse anti-MR antibody (gift from E. Gomez-Sanchez, Jackson, MS, USA) has been validated extensively in rat [23,25,26] and human cardiac tissues [27].

### 2.5. Epidural Steroid Injections (ESIs) for Alleviating Chronic Pain

Clinical use of corticosteroids in the epidural space has a long history [14], and substantial evidence supports the efficacy of epidural administration of specific glucocorticoid receptor (GR) agonists (e.g., triamcinolone, dexamethasone), either alone or in combination with local anesthetics [28]. In a previously published prospective, randomized controlled trial (RCT) [29], *n* = 52 patients with chronic lumbosacral radicular pain received an epidural injection consisting of 0.08% levobupivacaine combined with 40 mg triamcinolone. The primary aim of this study was to compare two treatment groups: one group received the injectate exclusively via the transforaminal route (3 mL), whereas the comparator group received the same transforaminal injection supplemented by an additional caudal epidural injection (10 mL of 0.025% levobupivacaine plus 40 mg triamcinolone). This design allowed evaluation of a potential synergistic effect between the two routes of administration. The primary outcome was the difference in patient-reported pain intensity between the two treatment regimens. Pain intensity was assessed using a 0–100 numeric rating scale (0 = no pain; 100 = worst imaginable pain) at baseline and at 1, 3, and 6 months after epidural treatment. The trial dataset and methodological details have been published previously [28]. In the present analysis, the two groups were not analyzed separately, as both examined the effects of epidurally administered triamcinolone; instead, a secondary analysis of all data was performed to assess changes in patient-reported pain intensity over time, from baseline to 6 months after treatment.

### 2.6. Statistics

All statistical analyses were performed using SigmaStat 2.03 (Systat Software GmbH Erkrath, Germany) for animal experiments and SPSS Version 29 (IBM Corporation, 2022, Armonk, NY, USA) for analyses of human data. In animals, paw pressure thresholds measured before and after drug administration were expressed as mean ± SD and evaluated using repeated-measures ANOVA followed by Dunnett’s post hoc test. For the secondary analysis of clinical data from 52 patients, raw data were extracted from the published dataset [28], and pain intensity scores were visualized using box plots across all time points. Differences in pain intensity from baseline to one, three, and six months were tested using stepwise Wilcoxon signed-rank tests. All statistical tests were two-sided with an alpha level of 0.05 and were considered exploratory.

## 3. Results

### 3.1. Relative Expression of GR mRNA Transcripts Compared to Transcripts of Pain-Associated Ion Channels, Neuropeptides, and MR in Human and Rat DRG

**Figure 1:**
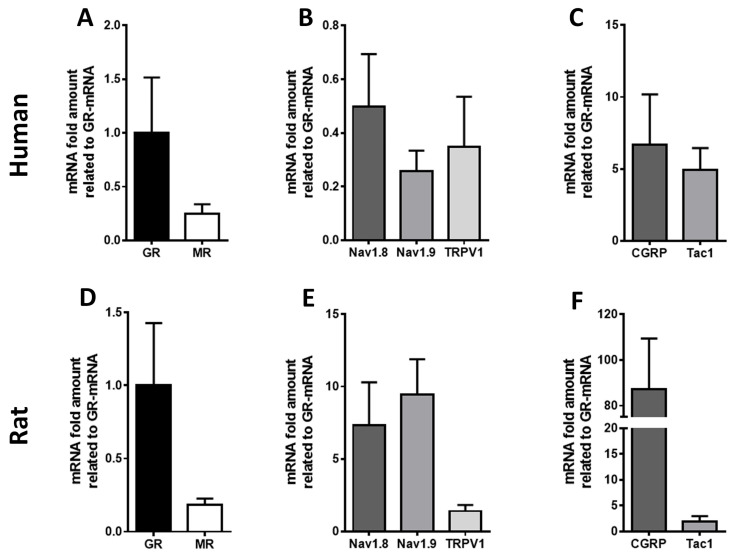
Detection of glucocorticoids receptor (GR), mineralocorticoid receptor (MR) (**A**,**D**), and key pain signaling molecule mRNA transcripts (**B**,**C**,**E**,**F**) in human (*n* = 4) (**A**,**B**,**C**) and rat (*n* = 5) (**D**,**E**,**F**) DRG sensory neurons. Quantitative TaqMan^®^ PCR confirmed the expression of GR and MR mRNA transcripts in human DRG (**A**), with Ct values of 21 and 23, respectively. In addition, TaqMan^®^ PCR analyses of human DRG tissue revealed that the mRNA expression levels of Nav1.8, Nav1.9, and TRPV1 were approximately in line with that of GR (**B**). In contrast, the mRNA levels of the neuropeptide CGRP and substance P (Tac1) were considerably higher than that of GR (**C**).

Using highly specific primer pairs, TaqMan^®^ qPCR detected mRNA expression of the corticosteroid receptors GR and MR in human DRG (Figure 1). GR mRNA levels were markedly higher than MR in both human and rat DRG (Figure 1). mRNA transcripts of typical pain-related ion channels (Nav1.8, Nav1.9, TRPV1) as well as of pain-associated neuropeptides (CGRP, Tac-1) were expressed in the same human and rat DRG; however, their expression was either comparable or higher relative to the expression of GR (Figure 1).

### 3.2. Immunohistochemical Demonstration of GR Protein in Colocalization with Typical Markers for Nociceptive Neurons in Human and Rat DRG

**Figure 2:**
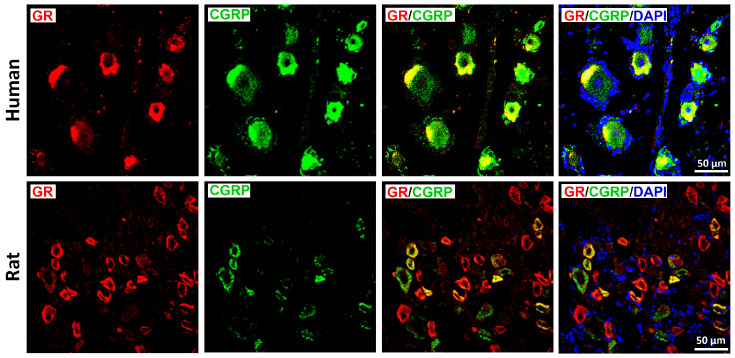
Identification of the glucocorticoids receptors (GRs) in human (upper panel) and rat (lower panel) DRG sensory neurons with the sensory neuronal marker CGRP. GRs were visualized by a specific antibody (see Table S3) and appropriate secondary antibody detected with red fluorescence, while CGRP was identified by green fluorescence. Note, not all CGRP-IR neurons contain MR immunoreactivity, and conversely, subset of MR-IR neurons showed no CGRP immunoreactivity. Nuclei were labeled with 4DAPI (bright blue). Bars represent 50 μm.

Double immunohistochemistry with a well-validated GR antibody [9,12,18] and fluorescence confocal microscopy revealed distinct GR immunoreactivity in human and rat DRG neurons, predominantly within the small diameter CGRP-IR neuron population (Figure 2). Some CGRP-IR neurons lacked GR, and vice versa.

**Figure 3:**
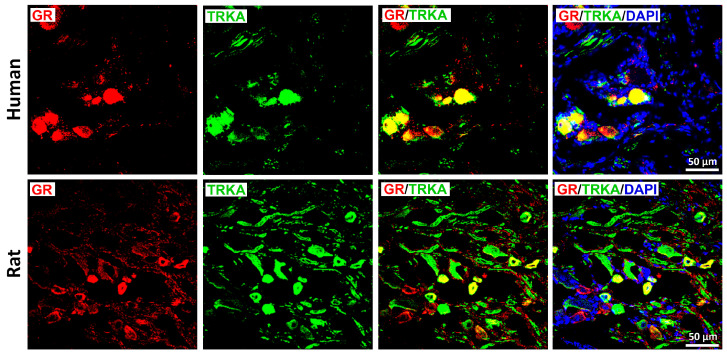
Detection of the glucocorticoids receptors (GRs) with the neurotrophic tyrosine kinase receptor (TrkA) in human versus rat sensory neurons within DRG. GRs are immunostained with red fluorescence, while TrKA are detected by green fluorescence. Note, GR-IR neurons exhibit a prominent colocalization with the key pain-signaling molecule TrkA in human (upper panel) and rat (lower panel) DRG neurons. Nuclei were stained with DAPI (bright blue). Bars represent 50 μm.

**Figure 4:**
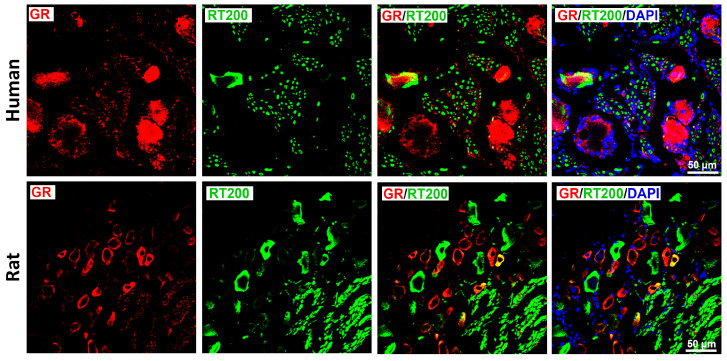
Detection of the glucocorticoids receptors (GRs) with the marker for myelinated A-type neurons neurofilament 200 (RT-200) in human (upper panel) versus rat (lower panel) sensory neurons of DRG. GRs were immunolabeled by specific antibody (see Table S3) and appropriate secondary antibodies detected with red fluorescence, while the RT-200 was identified by green fluorescence. A limited colocalization of GR with RT-200 represented by yellow fluorescence. Nuclei were identified with DAPI (bright blue). Bars represent 50 µm.

GR immunoreactivity was also enriched in peptidergic, nociceptive TrkA-IR C-type neurons in both species (Figure 3), although incomplete overlap was observed in both directions. In contrast, only limited colocalization was detected between GR and RT-200, a marker of myelinated A-type neurons (Figure 4); this was consistent with the small subset of rat DRG neurons exhibiting GR/RT200 co-labeling [9,12]. Rat DRG neurons were generally smaller than human DRG neurons when imaged at identical magnification (Figure 2).

**Figure 5:**
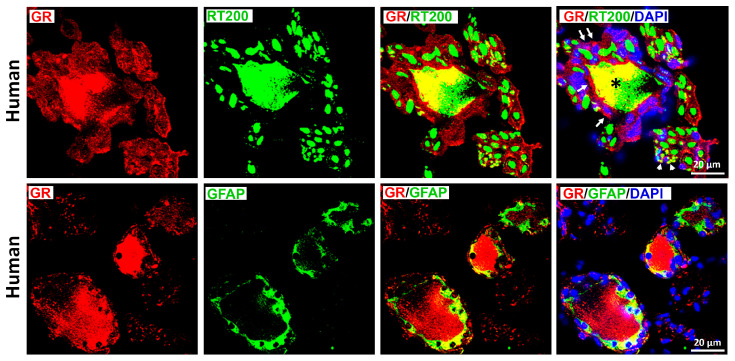
Identification of the glucocorticoids receptors (GRs) with the neurofilament 200 (RT-200) (upper panel) or satellite glial marker GFAP (lower panel) in human sensory DRG neurons. GR labeled with red fluorescence, while the RT-200 or GFAP visualized by green fluorescence. GRs were colocalized with RT-200-IR neuronal cell bodies (*) as well axons (white arrowhead) and in structures resembling satellite glial cells around the neurons (white arrows) (upper right panel). Note, GRs were colocalized with GFAP-IR satellite glial cells around the neurons expressing GR (red fluorescence). Note, GFAP-IR satellite glial cells around some neurons lacking GR immunoreactivity. Note, nuclei were detected with DAPI (bright blue). Bars represent 20 µm.

Overall, these findings demonstrate that GR is predominantly expressed in peptidergic CGRP- and TrkA-positive nociceptors, with minimal expression in myelinated RT200-positive neurons. Notably, GR immunoreactivity was also observed in cells morphologically consistent with satellite glial cells (Figure 4), which was confirmed by clear GR colocalization with the glial marker GFAP (Figure 5).

**Figure 6:**
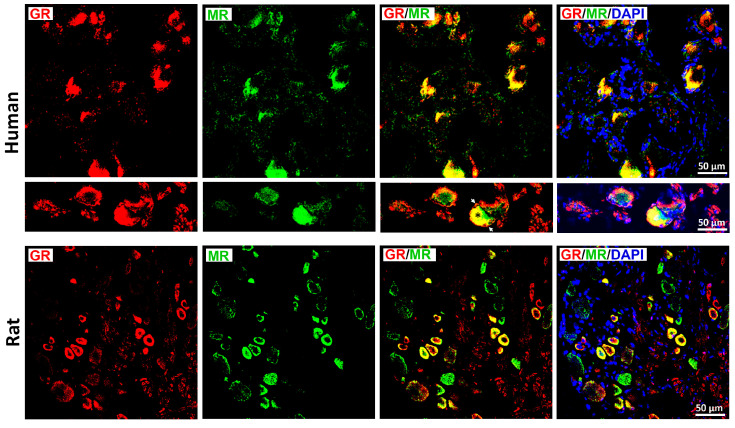
Detection of the glucocorticoids receptors (GRs) with mineralocorticoid receptors (MRs) in human (upper panel) versus rat (lower panel) DRG sensory afferent neurons. GRs are labeled with red fluorescence, while MRs are detected by green fluorescence. Note, the majority of GR-IR neurons contained MR immunoreactivity in both human (upper panel) and rat (lower panel) DRG. Additional image detail (middle panel) shows an example of GR with MR overlap (yellow) in which red fluorescence GR-immunostaining is also located in satellite glial cell-like structures (white arrowheads) around the DRG neurons (asterisk). Nuclei were counterstained with 4′,6-diamidino-2-phenylindole (DAPI; bright blue). Bars represent 50 μm.

**Figure 7:**
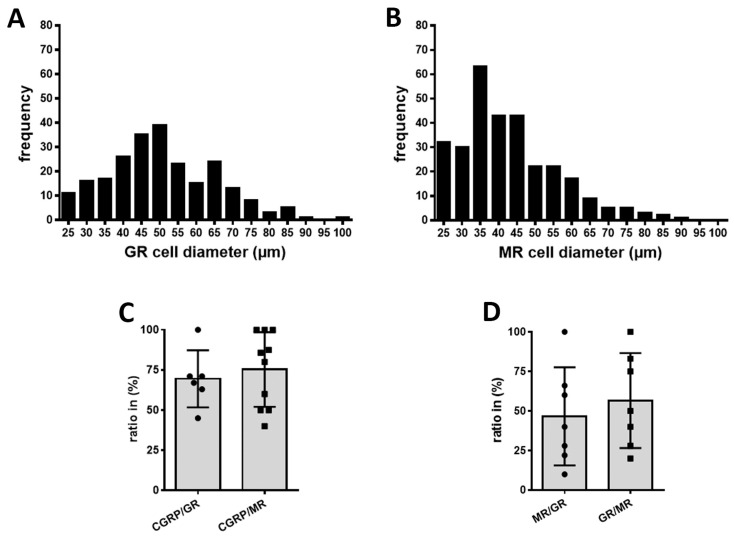
Semiquantitative analysis of human DRG neurons: size distributions of GR- and MR-immunoreactive (IR) neurons (**A**,**B**), co-expression of CGRP with GR or MR (**C**), and reciprocal GR/MR colocalization (**D**) in human DRG neurons. (**A**,**B**) MR-IR neurons were enriched among small- to medium-diameter cells (20–60 µm); this is characteristic of a nociceptor-like profile. In contrast, GR-IR neurons were most prevalent in medium-diameter cells (40–65 µm). Approximately 69% of CGRP-IR neurons co-expressed GR immunoreactivity and 75% co-expressed MR-IR, indicating predominant localization of both receptors in sensory (nociceptive) neurons. (**D**) Among MR-IR neurons, 46% co-expressed GR immunoreactivity; conversely, 56.6% of GR-IR neurons co-expressed MR immunoreactivity. Values are means ± SD.

Double immunofluorescence confocal microscopy demonstrated prominent colocalization of GR and MR in human DRG neurons (Figure 6, upper panel), which was consistent with observations in rat DRG (Figure 6, lower panel). First quantitative analyses of human DRG samples (*n* = 13–20) showed that MR-IR neurons were primarily small- to medium-diameter cells (25–60 µm), consistent with a nociceptive phenotype, whereas GR-IR was detected predominantly in medium-diameter neurons (40–65 µm) (Figure 7A–C). Further analysis revealed robust colocalization of both receptors with the sensory neuronal marker CGRP (CGRP/GR: 69.5  ±  7.3%; CGRP/MR: 75.3  ±  7.4%). The proportion of MR-IR neurons colocalizing with GR immunoreactivity and vice versa was 46.6  ±  11.7% (MR/GR) and 56.6  ±  11.3% (GR/MR), respectively (Figure 7).

### 3.3. GR- Versus MR-Mediated Modulation of Nociception in Rats with Inflammatory Pain

**Figure 8:**
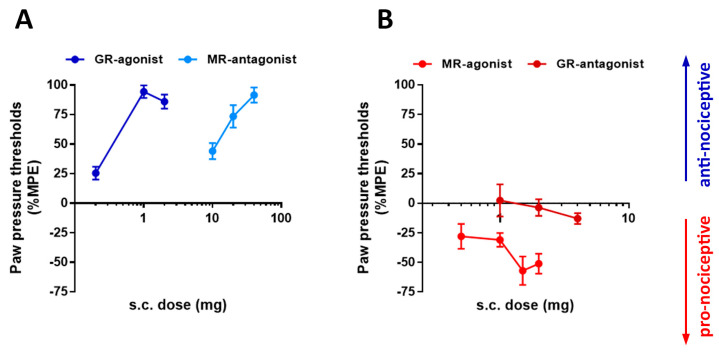
Effects of selective activation or blockade of GR and MR with specific agonists dexamethasone and aldosterone or antagonist mifepristone and canrenoate K, respectively, on nociceptive behavior evoked by Freund’s complete adjuvant (FCA) inflammation. (**A**) Increasing s.c. doses of GR agonist dexamethasone (Dexa) (0.1 and 0.2 mg/kg) as well as MR antagonist canrenoate K (Can) (10–40 mg/kg) produce significant increases in paw pressure threshold (PPT) relative to baseline, indicating attenuation of FCA inflammation-induced mechanical hyperalgesia (antinociceptive, blue) (*p* < 0.05, one-way ANOVA, followed by Bonferroni’s test for multiple pairwise comparisons, *n* = 6–8). (**B**) In contrast, increasing doses of s.c. MR agonist aldosterone (Aldo) (0.5–2 mg/kg) produced significant decrease in PPT relative to baseline (0 min), indicating an additional lowering of the already diminished thresholds for mechanically evoked pain (pronociceptive, red)(*p* < 0.05, one-way ANOVA, followed by Bonferroni’s test for multiple pairwise comparisons, *n* = 6), while GR antagonist mifepristone (1.0–4.0 mg/kg) produce no significant change in PPT (also pronociceptive, red) (*p* > 0.05. Data are expressed as means ± SD, *n* = 6–8). Dose ranges were selected based on pilot experiments.

FCA-induced inflammation of the right hind paw produced robust mechanical hypersensitivity in the inflamed, but not the contralateral hind paw (PPT values control versus inflamed: 70 ± 2.9 g vs. 50 ± 2.5 g). This inflammation-induced hypersensitivity was dose-dependently reversed by the GR-selective agonist dexamethasone and by the MR antagonist canrenoate K (spironolactone) (Figure 8). In contrast, the MR-selective agonist aldosterone further decreased mechanical withdrawal thresholds, which is consistent with a pronociceptive effect (Figure 8). The GR antagonist mifepristone alone did not significantly alter mechanical sensitivity (Figure 8).

### 3.4. Epidural Steroid Injections (ESIs) for Chronic Back Pain in Humans

**Figure 9:**
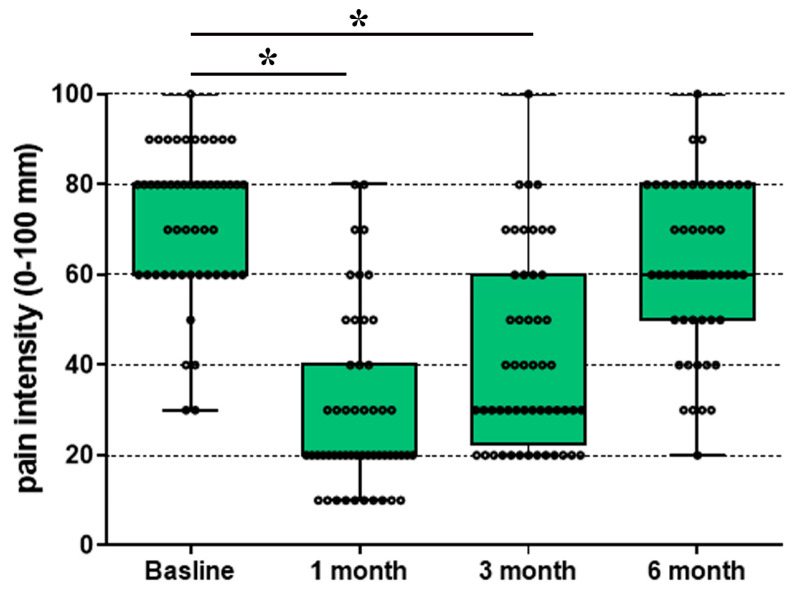
Time course of pain relief after epidural triamcinolone (GR agonist) with local anesthetic in chronic radicular pain. Fifty-four patients received a combination of transforaminal and caudal epidural injections of triamcinolone combined with a local anesthetic. Pain intensity, assessed on a standardized numerical rating scale (NRS), decreased from baseline to 1 month by median 66% (* *p* < 0.001) and after three months by median 50% (* *p* < 0.001) from baseline (Wilcoxon signed-rank test), whereas NRS scores retuned toward baseline by 6 months, indicating a sustained but time-limited analgesic effect. The early improvement is consistent with local anesthetic-mediated rapid analgesia, whereas the longer-lasting component likely reflects GR-dependent anti-inflammatory actions and stabilization of neuronal excitability. Data are median plus IQR (interquartile range).

Triamcinolone is a predominantly steroidal GR-selective agonist that mimics endogenous glucocorticoids and exerts potent anti-inflammatory and immunosuppressive effects [30]. We performed a secondary analysis of previously published clinical trial of *n* = 52 patients with chronic low back pain who received a single epidural triamcinolone injection in combination with local anesthetics. A significant reduction in pain intensity was observed over time (Figure 9). Numerical rating scale (NRS) scores decreased from baseline to 1 month by median 66% and after three months by median 50% from baseline pain intensity. Therefore, clinically meaningful analgesia persisted for up to 3 months, whereas pain ratings returned slowly to baseline by 6 months, indicating a sustained but time-limited therapeutic effect.

## 4. Discussion

Clinical evidence indicates that adding dexamethasone to local anesthetics in the perioperative setting significantly prolongs postoperative analgesia and reduces rebound pain as the anesthetic effect subsides [31,32]. These effects occur with both systemic and perineural administration of dexamethasone; however, lower doses are required perineurally, with longer-lasting analgesia [31,33], suggesting a localized action on peripheral sensory neurons. In this study, we identified GR mRNA and protein expression in human DRG neurons as potential mediators of this effect, which is consistent with findings in rat tissue. Double immunofluorescence revealed strong GR colocalization with CGRP-positive nociceptive neurons (~70–75%), occasional expression in satellite glial cells, partial overlap with mineralocorticoid receptor (MR, ~47–57%), and minimal presence in myelinated RT200-immunoreactive neurons. In a rat model of persistent localized inflammatory pain, GR agonists reduced, while MR agonists exacerbated, nociceptive responses suggesting opposing roles in pain modulation. Notably, MR antagonists mirrored the analgesic effects of GR agonists. Clinically, long-acting GR agonists such as triamcinolone have demonstrated durable analgesia in patients with chronic radicular lumbosacral pain. Taken together, these findings align with clinical experience showing that long-acting GR agonists can provide sustained but ultimately time-limited pain relief, and they support a mechanistic role for perineural GR activation in prolonging analgesia.

Our findings are consistent with recent work by [17], who employed RNAscope in situ hybridization to visualize GR and MR transcripts as discrete puncta at the single-cell level in human DRG. In their study, GR and MR mRNA signals were detected in nearly all DRG neurons in both mice and humans, with GR and MR colocalization reported in up to 99% of neurons. In contrast, our results show a clear predominance of GR over MR transcript expression (~5-fold), accompanied by only partial (~50%) colocalization of GR and MR protein immunoreactivity in DRG neurons. This discrepancy may reflect species-specific or methodological differences, or differential post-transcriptional regulation of these receptors.

Using double-label confocal immunofluorescence, we identified predominant colocalization of GR and MR with the peptidergic nociceptor marker CGRP in a subset of human DRG neurons, mirroring patterns previously reported in rat DRG. These findings provide compelling evidence that GR and MR are enriched in CGRP-IR human DRG afferents, extending earlier observations of predominant GR/MR expression in CGRP-positive rat sensory neurons [9,12,18] and of corticosteroid receptors at peripheral nerve terminals in human skin [34]. Notably, GR immunoreactivity was also detected in SGC surrounding human DRG neurons, implicating glia-mediated corticosteroid signaling. This observation is consistent with prior rodent studies reporting GR expression in DRG as well as SGC and linking GR/MR signaling to glial activation [35]. Collectively, these findings suggest a conserved, peptidergic nociceptor-biased organization of corticosteroid receptors in human DRG and underscore the need to further dissect receptor- and cell type-specific mechanisms as potential targets for analgesia.

We next examined how selective activation of GR versus MR modulates inflammatory pain. Administration of the GR agonist dexamethasone produced a dose-dependent attenuation of FCA-evoked nociception within 15 min, without altering sensitivity in the contralateral, noninflamed paw. The rapid onset and activity dependence of this effect are consistent with a nongenomic GR mechanism that preferentially engages under conditions of ongoing nociceptor activity. This interpretation aligns with earlier reports demonstrating that glucocorticoids suppress spontaneous ectopic firing in neuromas, inhibit ATP-evoked DRG currents, and reduce C-fiber conduction, while exerting minimal effects in quiescent neurons [16,24,36].

In contrast, the MR agonist aldosterone acutely exacerbated mechanical hypersensitivity in the inflamed paw within the same short time frame. This pronociceptive action is in line with in vitro findings in acutely dissociated DRG neurons from naïve rats, where incubation with aldosterone (peak effect at ~10 nM) increased the number of action potentials elicited by suprathreshold current injections [7,37]. These results parallel prior in vivo work in models of dorsal root compression and inflammation, showing that the MR antagonist eplerenone reduced hyperalgesia and neuronal excitability, whereas aldosterone enhanced action-potential firing during local inflammation and suprathreshold DRG stimulation [7,37]. Consistent with this, pharmacological blockade of MR with canrenoate K rapidly normalized FCA-induced mechanical hypersensitivity in our animal pain model.

Together, these findings support opposing, activity-dependent roles of corticosteroid receptors in primary sensory pathways: GR activation suppresses, whereas MR activation amplifies, inflammatory nociception. Moreover, they raise the possibility that an endogenous MR agonist may drive pronociceptive signaling in inflamed tissue via sensory neuronal MR [38].

Clinically, the analgesic use of neuraxial corticosteroids (e.g., intrathecal or epidural), as reviewed by Davis (2010) [39] and Pertovaara and Breivik (2016) [40], underscores the functional relevance of corticosteroid signaling within human sensory pathways. Consistent with this, in our secondary analysis of previously published clinical trial data, epidural administration of triamcinolone in combination with local anesthetics in patients with radicular lumbar pain resulted in a significant, time-dependent reduction in pain intensity (Figure 9). The combined treatment likely produced rapid analgesia through the local anesthetic component and more prolonged pain relief through GR-mediated anti-inflammatory and neuronal-stabilizing mechanisms. These observations support a complementary and potentially synergistic interaction between acute nociceptive blockade and GR activation. Moreover, the predominant expression of GR in human DRG demonstrated in our study aligns with an expanding body of clinical literature documenting the successful intrathecal use of glucocorticoids for neuropathic pain syndromes, particularly sciatica and postherpetic neuralgia, dating back to the early reports by [41].

This study has several important limitations. First, the human dataset consisted of only two donors (one male, one female), limiting robust quantification of DRG neuronal subtypes and generalizability as well as constraining meaningful cross-species comparisons with rat DRG. Second, although GR and MR share ~95% amino-acid identity between rat and human, antibody specificity may still differ across species; this concern is amplified by the fact that fixation of the commercially sourced human DRG occurred outside our control, potentially affecting epitope integrity. Third, all rat samples were male, whereas the human samples included both sexes, introducing potential sex-related confounds. Fourth, we did not perform a comprehensive, large-scale mapping of GR and MR expression in human DRG; such efforts will require larger cohorts and deeper phenotypic resolution. Finally, the two human DRG samples were obtained from young fatal-accident donors, and factors such as age, prior pain history, or medication exposure may influence receptor expression and limit generalizability. Accordingly, this work should be interpreted as an initial immunohistochemical characterization of GR and MR in human versus rat DRG.

## 5. Conclusions

In conclusion, this study demonstrates that glucocorticoid signaling is a prominent molecular feature of human and rat DRG nociceptors, with GR expressed at markedly higher levels than MR and enriched within peptidergic CGRP- and TrkA-positive neurons as well as a subset of satellite glial cells. Functionally, GR activation rapidly attenuated inflammatory nociception, whereas MR activation exacerbated it, revealing opposing, activity-dependent roles for these receptors in peripheral pain processing. The efficacy of epidural triamcinolone in patients with chronic radicular pain further supports a clinically relevant analgesic pathway mediated by GR. Together, these molecular, functional, and clinical findings identify GR as a key modulator of sensory neuron excitability and pain, highlight MR as a pronociceptive counterpart, and suggest that selectively enhancing GR signaling or inhibiting MR signaling may offer a mechanistically grounded strategy for improving corticosteroid-based analgesic therapies.

## Data Availability

Data can be accessed upon request by contacting the first author via e-mail: shaaban.mousa@charite.de.

## Supplementary Materials

The following supporting information can be downloaded at https://www.mdpi.com/article/10.3390/cells15030223/s1, Supplementary Table S1: List of Primers for Taqman RT-PCR of human DRG; Supplementary Table S2: List of Primers for Taqman RT-PCR of rat DRG, Supplementary Table S3: Characterization of primary antibodies used, Supplementary Figure S1 Immunofluorescence staining of human dorsal root ganglia tissue using Alexa Fluor 594 donkey anti-rabbit antibody (Texas red immunofluorescence) and Alexa Fluor 488 goat anti-mouse antibody (FITC green fluorescence) as secondary antibodies with omission of the respective primary antibodies (blank control). Nuclei were counterstained with 4′,6-diamidino-2-phenylindole (DAPI; bright blue). Scale bar = 40 μm.
