# Supplementary material for: Human DRG Glucocorticoid Receptor Profiling Reveals Targets for Regionally Delivered Steroid Analgesia
Source: Cells. 2026 Jan 24;15(3):223. doi: 10.3390/cells15030223 (PMC12896501; doi:10.3390/cells15030223)

## Table S1: List of Primers for Taqman RT-PCR of human DRG

| Gene | Access. Nr. | Forward | Reverse |
|---|---|---|---|
| **GR** | NM_0001762 | 5'-tgctccttctgcgttcacaa-3' | 5'-ccatcagtgaatatcaactctggc-3' |
| **MR** | NM_000901.4 | 5'-aaagagcagtggaagggcaa-3' | 5'-tgaagtctgcaagcaggacaa-3' |
| **Nav.1.8** | NM_006514.4 | 5'ctccgtccttgaggaactcg-3 | 5'-tctgcaaagggatccgtcac-3' |
| **Nav.1.9** | NM_001349253.2 | 5'agccaggaatctcgggtgaa-3 | 5'-agccagagagtcggaagtga-3' |
| **TRPV1** | NM_080704.4 | 5'-gcaggacaagtgggacagat-3' | 5'-tcttaaagggaggcaagcca-3' |
| **CGRP** | NM_001378950.1 | 5'-actggtgcaggactatgtgc-3 | 5'-actggtgcaggactatgtgc-3' |
| **Tac1** | NM_003182.3 | 5'-aagtggccctgttaaaggct-3 | 5'-gcactcctttcataagccacaga-3 |
| **S18** | NR_046237 | 5'-cggctaccacatccaaggaa-3' | 5'-cctggaattaccgcggct-3' |

**Table S2: List of Primers for Taqman RT-PCR of rat DRG**

| Gene | Access. Nr. | Forward | Reverse |
|---|---|---|---|
| **GR** | NM_012576.2 | 5’-catcttcagaacagcaaaatcga-3’ | 5’-aggtgctttggtctgtgggata-3’ |
| **MR** | NM_013131.1 | 5’-ccaaggtacttccaggatttaaaaac-3’ | 5’-aacgatgatagacacatccaagaatact-3’ |
| **Nav.1.8** | NM_017247.1 | 5’-cacggatgacaacaggtcac-3’ | 5’- gatcccgtcaggaaatgaga-3’ |
| **Nav.1.9** | NM_019265.2 | 5’-ccaggagccttgtttcccat-3 | 5’-tttatgcacagccactgagg-3’ |
| **TRPV1** | NM_031982 | 5’-agtgagacccctaaccgtca-3’ | 5’-cggaaatagtccccaacggt-3’ |
| **CGRP** | NM_001033956.1 | 5’-cctttcctggttgtcagcatctt-3’ | 5’-cagtaggcgagcttcttcttcac-3’ |
| **Tac1** | NM_012667 | 5’-cacccgatacctccagacaca-3’ | 5’-ggagccgttggaggtgaga-3’ |
| **18S** | NR_046237.2 | 5’cggctaccacatccaaggaa-3 | 5’-gctggaattaccgcggct-3’ |

**Table S3: Characterization of primary antibodies used**

| Antigen | Immunogen | Manufacturer, Species, Type, Catalogue Number | Dilution used |
|---|---|---|---|
| GR | A part of the rat GR transcription modulation domain | A gift from M. Kawata (Kyoto Prefectural University of Medicine, Japan), polyclonal [19] | 1:3.000 |
| MR | generated against epitopes located at the N terminal of the MR molecule | Elise Gomez-Sanchez, Jackson, USA, rMR 79–87 monoclonal antibody [23] | 1.200 |
| CGRP | synthetic entire calcitonin gene-related peptide | Peninsula Laboratories (CA, USA), guinea pig polyclonal, # T-5027 [20] | 1:1.000 |
| trkA | Extracellular domain Ala33-Pro418 of rat trkA | R&D Systems (USA), goat polyclonal, # AF1056 [21] | 1:500 |
| NF200 | Carboxy terminal tail segment of dephosphorylated NF200 | Sigma-Aldrich (USA), mouse monoclonal #N0142/N52 [22] | 1:1.000 |
| GFAP | clone G-A-5 | Sigma-Aldrich (USA), mouse monoclonal #G3893 [24] | 1:1.000 |

## References

19. Ito, T.; Morita, N.; Nishi, M.; Kawata, M. In vitro and in vivo immunocytochemistry for the distribution of mineralocorticoid receptor with the use of specific antibody. Neuroscience research 2000, 37, 173-182, doi:10.1016/s0168-0102(00)00112-7.

20. Mousa, S.A.; Hong, X.; Metwally, E.Y.; Tafelski, S.; Wandrey, J.D.; Piontek, J.; Treskatsch, S.; Schäfer, M.; Shaqura, M. Components of Mineralocorticoid Receptor System in Human DRG Neurons Co-Expressing Pain-Signaling Molecules: Implications for Nociception. Cells 2025, 14, doi:10.3390/cells14151142.

21. Matsumoto, K.; Suenaga, M.; Mizutani, Y.; Matsui, K.; Yoshida, A.; Nakamoto, T.; Kato, S. Role of transient receptor potential vanilloid subtype 2 in lower oesophageal sphincter in rat acid reflux oesophagitis. J Pharmacol Sci 2021, 146, 125-135, doi:10.1016/j.jphs.2021.03.010.

22. Kestell, G.R.; Anderson, R.L.; Clarke, J.N.; Haberberger, R.V.; Gibbins, I.L. Primary afferent neurons containing calcitonin gene-related peptide but not substance P in forepaw skin, dorsal root ganglia, and spinal cord of mice. The Journal of comparative neurology 2015, 523, 2555-2569, doi:10.1002/cne.23804.

23. Gomez-Sanchez, C.E.; de Rodriguez, A.F.; Romero, D.G.; Estess, J.; Warden, M.P.; Gomez-Sanchez, M.T.; Gomez-Sanchez, E.P. Development of a panel of monoclonal antibodies against the mineralocorticoid receptor. Endocrinology 2006, 147, 1343-1348, doi:10.1210/en.2005-0860.

24. Liu, L.; Chen, X.L.; Yang, J.K.; Ren, Z.G.; Wang, S. Gliosis after traumatic brain injury in conditional ephrinB2-knockout mice. Chin Med J (Engl) 2012, 125, 3831-3835.

## Supplemental Figure S1:

Immunofluorescence staining of human dorsal root ganglia tissue using Alexa Fluor 594 donkey anti-rabbit antibody (Texas red immunofluorescence) and Alexa Fluor 488 goat anti-mouse antibody (FITC green fluorescence) as secondary antibodies with omission of the respective primary antibodies (blank control). Nuclei were counterstained with 4′,6-diamidino-2-phenylindole (DAPI; bright blue). Scale bar = 40 µm.

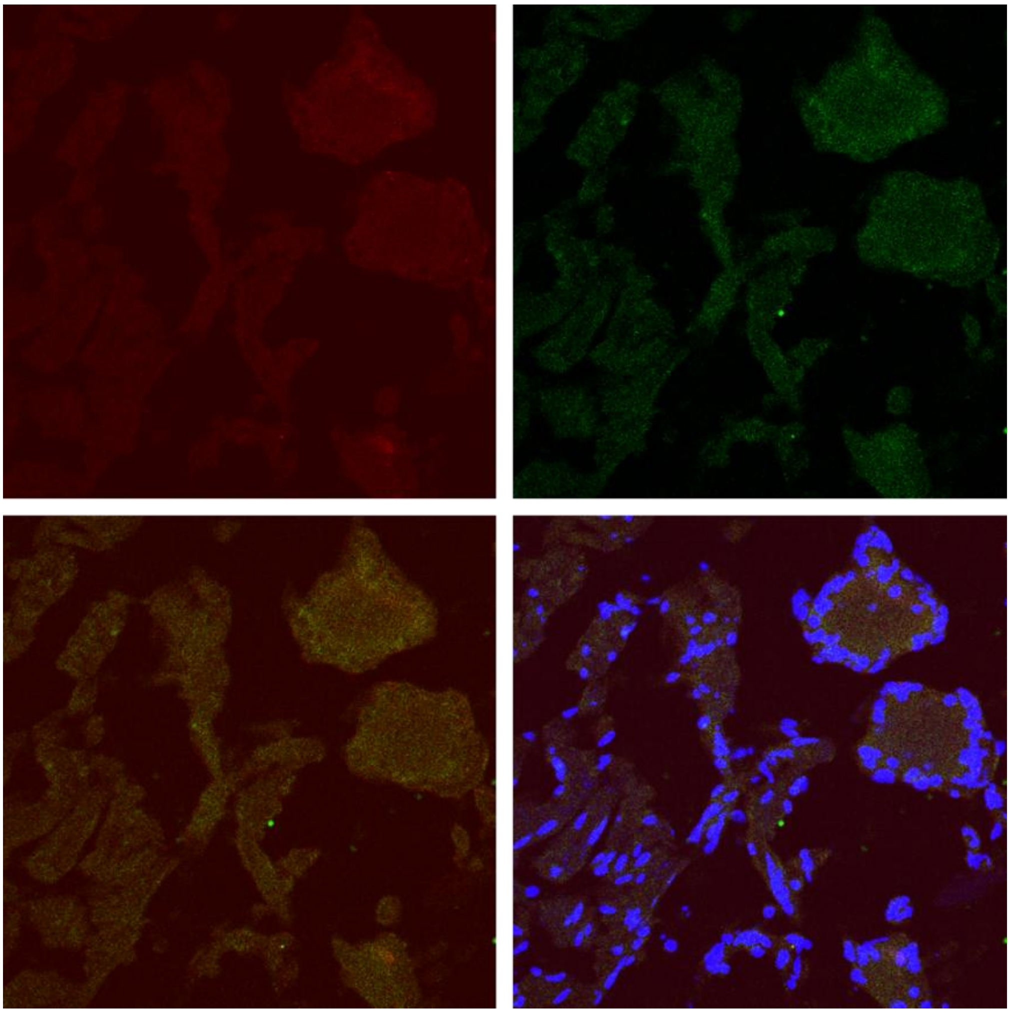

Supplement: Supplementary file 1 [file cells-15-00223-s001.zip › cells-4061595-supplementary.pdf]
